# A global deep terrestrial biosphere core microbiome

**DOI:** 10.1093/ismeco/ycaf176

**Published:** 2025-10-07

**Authors:** Carolina González-Rosales, Maryam Rezaei Somee, Moritz Buck, Stefan Bertilsson, Maliheh Mehrshad, Mark Dopson

**Affiliations:** Center for Ecology and Evolution in Microbial Model Systems (EEMiS), Linnaeus University, Universitetsplatsen 1, 392 31 Kalmar, Sweden; Center for Ecology and Evolution in Microbial Model Systems (EEMiS), Linnaeus University, Universitetsplatsen 1, 392 31 Kalmar, Sweden; Department of Aquatic Sciences and Assessment, Science for Life Laboratory, Swedish University of Agricultural Sciences, 750 07 Uppsala, Sweden; Department of Aquatic Sciences and Assessment, Science for Life Laboratory, Swedish University of Agricultural Sciences, 750 07 Uppsala, Sweden; Department of Aquatic Sciences and Assessment, Science for Life Laboratory, Swedish University of Agricultural Sciences, 750 07 Uppsala, Sweden; Center for Ecology and Evolution in Microbial Model Systems (EEMiS), Linnaeus University, Universitetsplatsen 1, 392 31 Kalmar, Sweden

**Keywords:** groundwater, microbial ecology, microbiome, metagenomics

## Abstract

The deep biosphere encompasses life beneath the Earth’s surface and constitutes a substantial portion of the planet’s microbial biomass. This study analyzed nucleic acid datasets from low-carbon and low-energy deep terrestrial subsurface groundwaters across four continents and revealed four core global populations. These populations exhibited metabolic strategies and adaptations reflecting depth and environmental constraints. *Erythrobacter* featured heterotrophic metabolism; *Thiobacillus* demonstrated sulfur oxidation coupled to denitrification along with carbon and nitrogen fixation; Methanobacteriaceae were methanogenic autotrophs using the Wood–Ljungdahl pathway (WL); and *Candidatus* Desulforudis audaxviator functioned as a sulfate-reducer also encoding the WL pathway. Depth-related adaptations suggested heterotrophic dominance at shallower depths with increasing contributions from autotrophy with depth. Finally, comparative genomics revealed minimal evolutionary changes among these populations, suggesting functional conservation since diverging from their ancestral lineages. These findings underscore a global deep biosphere core community.

## Introduction

The deep biosphere encompasses life in both terrestrial and marine subsurface environments (i.e. bedrock below the soil horizon [1] and sediment below the marine bioturbation zone [2]) and extends over several kilometers depth [3]. While extant deep life was first demonstrated in the marine subsurface [4], subsequent studies have revealed active prokaryotes [5–7], eukaryotes [8, 9], and viruses [10, 11] in globally distributed terrestrial groundwaters. Estimates suggest that the deep subsurface hosts circa 90% of the total bacteria and archaea biomass, accounting for ~10%–20% of the Earth’s total biomass [12, 13]. The Earth’s crystalline aquifers sustain vast and diverse microbial ecosystems [14], whose diversity is influenced by parameters such as temperature, pressure, water residence time, and geochemistry [15], with most cells predicted to be attached to rock surfaces [16].

Sites such as Olkiluoto Island (Finland) and the Äspö Hard Rock Laboratory (HRL; Sweden) are the most extensively studied terrestrial subsurface environments, and are included in the Fennoscandian Shield Genomic Database [17]. These datasets reveal microbial adaptation strategies to survive in low-energy conditions, including small cell size, streamlined genomes compared to surface taxa, a high functional interactivity such as syntropy aided by biofilm formation, and episodic lifestyles proceeding when energy is available [17–19].

The deep terrestrial subsurface microbial diversity has been studied in, for instance, borehole water and granite rock cores [20], subsurface aquifers [21], boreholes in sedimentary geological settings [22], and anoxic boreholes in Precambrian bedrock [23] with microbial distribution patterns structured according to the availability of energy and nutrients [17, 21]. Despite the impact of lithology and low resource availability in shaping the community in deep terrestrial subsurface ecosystems, a common core microbiome of 73 genome clusters (at species level with >95% average nucleotide identity, ANI) was detected for two disconnected deep Fennoscandian Shield groundwaters with traits pointing to a key role of biological interactions and energy efficient metabolism for their convergence [17]. In addition, a 16S rRNA gene-based survey of 233 subsurface samples in five countries (between 94 and 2300 m below the surface) revealed a dominance of Betaproteobacteria, Gammaproteobacteria, and Firmicutes in the communities, along with a core community formed by Betaproteobacteria and Gammaproteobacteria [19]. Another genome-resolved analysis detected *Candidatus* Desulforudis audaxviator in deep subsurface samples across three continents: South African gold mines (Africa), a Paleozoic carbonate aquifer in Nevada, California (North America), and a Siberian Cretaceous aquifer (Asia) [24, 25]. These *Candidatus* Desulforudis audaxviator genomes reveal a high degree of conservation manifested by high ANI, few single nucleotide polymorphisms, and conservation of prophages plus CRISPRs. This suggests minimal evolution since their separation from the ancestral population between ca. 165 and 55 Ma years ago [25]. Although recent advances have been made, understanding of the conserved microbial populations in the deep terrestrial subsurface remains limited and consequently, their evolutionary dynamics remain unknown.

This study examined over 4000 metagenome-assembled genomes (MAGs) and single-amplified genomes (SAGs) reconstructed from deep terrestrial subsurface ecosystems across four continents to test the hypothesis that there is a global core terrestrial deep biosphere microbiome adapted to low-carbon and -energy conditions. The deep terrestrial groundwater metagenomes were further compared to determine how different water types affected microbial community differentiation and the variation of these communities across different depths. In addition, this work significantly expands the previously established Fennoscandian Shield Genomic Database, originally focused on Scandinavian subsurface environments, by broadening the (meta)genomic resources available for investigating deep subsurface microbial diversity and biogeography on a global scale.

## Materials and methods

### Groundwater metagenomic datasets

Deep groundwater metagenomes were collected from the NCBI, JGI, and MG-RAST databases, which were supported by scientific publications. The metagenomes were selected based on a minimum sampling depth of 70 mbsl (Supplementary Tables 1 and 2), excluding data from environments such as oil, gas, landfill, and shale locations to focus on low-carbon and low-energy deep groundwater niches. SAGs from these groundwaters were selected according to the same criteria (Supplementary Table 3). The datasets were separated based on the sampling depth into three categories: (i) 70–999 mbsl, (ii) 1000–1999 mbsl, and (iii) ≥2000 mbsl.

Depth category (i) included samples from three continents (North America, Asia, and Europe) from groundwater in bedrocks at depths ranging from 95 to 129 mbsl in Alberta, Canada [26]; high pH aquifers from the serpentinizing ophiolite at the Coast Range Ophiolite Microbial Observatory, USA at 76 mbsl [27]; nitrate-rich groundwater at 100 mbsl in San Joaquin Valley, USA [28]; brine waters from boreholes at 715 mbsl in Soudan Underground Mine State Park, USA [29]; and samples from CO_2_ saturated groundwater erupted from Crystal Geyser (~200 to 800 mbsl), USA [30–34], which reflects the mixing of multiple aquifers accessed via an 800 m deep borehole. In Europe, samples were collected from 70 to 455 mbsl at the Äspö HRL, Sweden [17, 35, 36]; Olkiluoto Island, Finland at depths from 331 to 532 mbsl [37–39]; aquifers from the Iberian Pyrite Belt, Spain at around 400 mbsl [40]; and Opalinus Clay borehole water [5, 41] from 226 to 563 mbsl in Mont Terri, Switzerland. Representing the Asian continent, samples were collected from 140 to 250 mbsl at the Horonobe underground research laboratories (URL), Japan [42]. Category (ii) consisted of samples from the 1.8 km deep Cambrian-age Mt. Simon Sandstone deposits in the Illinois Basin, USA [43]; South African mine samples including Masimong at 1900 mbsl [44], Beatrix [44, 45], and Welkom area [46] at depths around 1340 mbsl; Finsch mine at a depth of 1056 mbsl [44]; and Driefontein mine at 1046 mbsl [44]. The deepest category (iii) samples were from the Thabazimbi area (2100 mbsl) [44]; TauTona gold mine at 3048 and 3136 mbsl [44, 47], and Mponeng [24] mines in South Africa. Additionally, samples were obtained from 2600 to 2800 mbsl in the Russian Tomsk region [21, 48, 49].

### Metagenome analysis

Detailed methods are given in the Supplementary material. Briefly, low-quality bases in the metagenome sequences were trimmed and filtered, assembled, mapped to their assemblies, contigs were binned plus quality tested, and de-replicated (Supplementary Table 4). The MAGs and SAGs were assigned taxonomy and phylogenomic trees were constructed with unclassified populations being selected for further analysis (Supplementary Table 5). Alpha (Supplementary Table 6) and beta diversity metrics were calculated. The relative abundance of subsurface genomes was calculated using transcripts per million (TPM) normalization and log_10_ transformation. Heatmaps were created to compare abundance by location to identify conserved populations. Taxa identified as potential contaminants were removed from the microbial populations based on genera most frequently associated with contamination in the Census of Deep Life Dataset [50]. Functional annotation and prediction of metabolic functions were performed. The *Candidatus* Desulforudis audaxviator genome was mapped onto metagenome dataset, core populations were searched, and these groups were compared using ANI and genome synteny analyses.

## Results and discussion

### The global groundwater microbiome dataset

**Figure 1:**
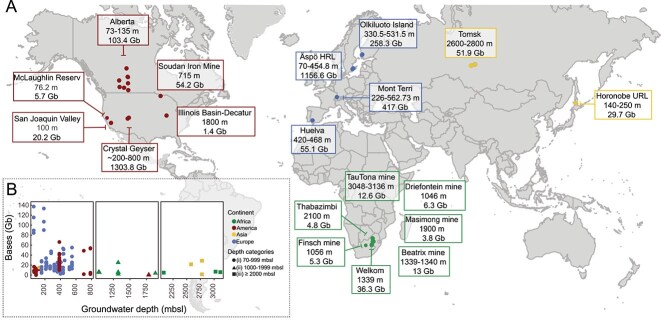
Distribution of worldwide oligotrophic deep groundwaters metagenomes. The box color for each location corresponds to different continents (A) North America is dark red, Europe is blue, Asia is yellow, and Africa is green. Depth ranges of the samples (indicated in red) and the amount of sequenced data (presented as gigabase pair, Gb) are shown for each location. Genomic information from the metagenomic dataset (presented in Gb) is distributed across three depth categories (B) 70–999 mbsl (●), 1000–1999 mbsl (▲), and ≥2000 mbsl (■). The colors of the shapes correspond to the continents based on sampling origin: America is dark red, Europe is blue, Asia is yellow, and Africa is green.

A total of 174 publicly available metagenomic datasets (Fig. 1A) originating from ≥70 m depth (Supplementary Tables 1 and 2) and 305 SAGs (Supplementary Table 3) were collected. The datasets were from 19 locations in nine countries (Canada, Finland, Japan, Russia, South Africa, Spain, Sweden, Switzerland, and the USA) across Africa, Asia, Europe, and North America. These datasets represent deep groundwater communities originating from planktonic samples in waters of a meteoric origin at 70 mbsl from the Äspö HRL, Sweden [35] down to boreholes intersecting water at 3136 mbsl from the TauTona gold mine in South Africa [44]. The total size of these metagenomes was 3563 giga base pairs, with the majority of the data from Crystal Geyser (USA), Äspö HRL, and Mont Terri, Switzerland (36.6%, 33.1%, and 11.7% of total base pairs, respectively). In contrast, datasets generated from the Thabazimbi area (South Africa), Masimong gold mine (South Africa), and Illinois Basin-Decatur (USA) had the smallest sizes, accounting respectively for 0.1%, 0.1%, and <0.1% of the sequence data. A total of 4451 MAGs were reconstructed from these datasets and the total 4756 MAGs/SAGs were filtered for completeness (≥50%) and contamination (≤5%) to yield 4102 MAGs and 125 SAGs. Dereplication at 95% ANI threshold resulted in 2255 genome clusters that characterize the available global deep groundwater microbiome (Supplementary Table 4).

The metagenomic datasets were separated based on the sampling depth into three categories: (i) 70–999 mbsl (*n* = 156), (ii) 1000–1999 mbsl (*n* = 12), and (iii) ≥2000 mbsl (*n* = 6); accounting for 96.2%, 1.9%, and 1.9% of the total genomic information (base pairs), respectively (Fig. 1B, Table 1). The low representation of metagenomic samples in the deeper categories (ii, iii) was likely attributed to the difficulties of obtaining uncontaminated samples from greater depths.

**Table 1:** Metagenomic data included in the global deep biosphere database and their depth classification.

**Location**	**Country**	**#MetaG**	**Gigabase pairs**	**Depth (m)**	**Depth category**			**Reference**
					(i)	(ii)	(iii)	
Alberta	Canada	8	103.4	73–135	8			Ruff *et al.* [26]
Olkiluoto Island	Finland	17	258.3	330.5–531.5	17			Bell *et al.* [37–39]
Horonobe	Japan	2	29.7	140–250	2			Hernsdorf *et al.* [42]
Tomsk	Russia	3	51.9	2600–2800			3	Kadnikov *et al*. [48, 49]
Beatrix mine	South Africa	3	13	1339–1340		3		Lau *et al.* [44], Harris *et al.* [45]
Driefontein mine	South Africa	1	6.3	1046		1		Lau *et al.* [44]
Finsch mine	South Africa	1	5.3	1056		1		Lau *et al.* [44]
Masimong mine	South Africa	1	3.8	1900		1		Lau *et al.* [44]
TauTona mine	South Africa	2	12.6	3048–3136			2	Lau *et al.* [44], Magnabosco *et al.* [47]
Thabazimbi area	South Africa	1	4.8	2100			1	Lau *et al.* [44]
Welkom, Witwatersrand Basin	South Africa	3	36.3	1339		3		Lau *et al.* [46]
Huelva, Peña de Hierro	Spain	2	55.1	420–468	2			Puente-Sánchez *et al.* [40]
Äspö HRL	Sweden	36	1156.6	70–454.8	36			Mehrshad *et al.* [17], Dopson *et al.* [35], Rezaei Somee *et al.* [36]
Mont Terri	Switzerland	18	417	226–562.73	18			Bagnoud *et al.* [5, 41]
San Joaquin Valley	USA	5	20.2	100	5			Ludington *et al.* [28]
McLaughlin Reserve	USA	1	5.7	76.2	1			Putman *et al.* [27]
Crystal Geyser	USA	63	1303.8	320–800	63			Probst *et al.* [30, 32, 33], Burstein *et al.* [31]
Basin-Decatur	USA	3	1.4	1800		3		Dong *et al.* [43]
Soudan Iron Mine	USA	4	54.2	715	4			Sheik *et al.* [29]

### The global groundwater microbial community

**Figure 2:**
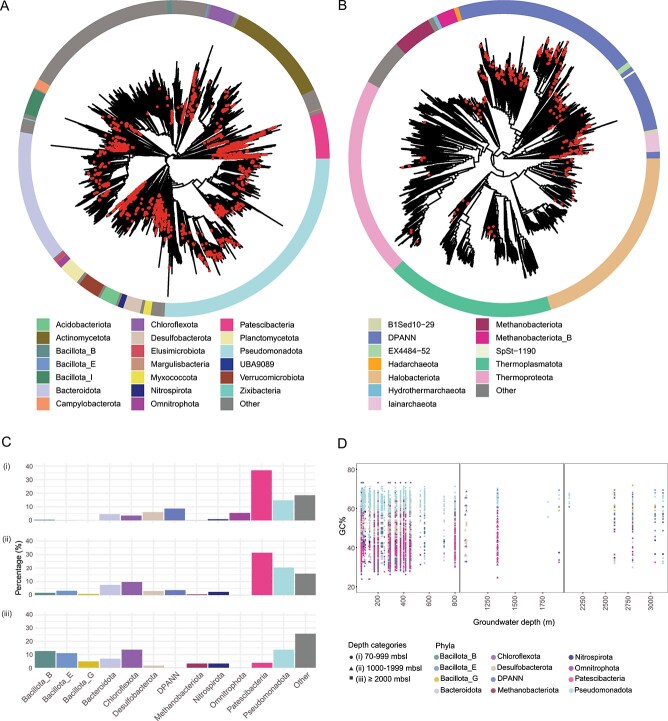
Global representatives from deep groundwaters showing their phylogeny, depth distribution, and GC content at differing depths. The bacterial (A) and archaeal (B) trees were generated using GTDB-Tk and visualized with R package ggtree with the genomes in the complete data set indicated by red dots. The bar chart (C) shows the percentage of representative MAGs and SAGs at the phylum level identified across the three depth categories of 70–999 mbsl (i, top), 1000–1999 mbsl (ii, middle), and ≥ 2000 mbsl (iii, bottom). (D) Distribution of the 2255 representatives for GC content within each depth category: 70–999 mbsl (●), 1000–1999 mbsl (▲), and ≥2000 mbsl (■). The colors of the shapes correspond to the phyla.

According to the GTDB [51, 52] classification, 85.5% of the community representatives were affiliated to domain Bacteria (*n* = 1927) and 14.5% to Archaea (*n* = 328; Supplementary Fig. 1A and B). This matches a previous meta-analysis of the deep biosphere microbiome [13] except for a few groundwaters such as Lidy Thermal Springs, USA containing >99% archaea [53]. Phylogenetic reconstruction showed that the global terrestrial deep biosphere representatives were broadly distributed across different phyla, suggesting that adaptation to the low-carbon and -energy conditions typical of the deep terrestrial biosphere has occurred within multiple taxonomic lineages. The majority of representative MAGs/SAGs were affiliated to the Patescibacteria (27.4%), Pseudomonadota (13.8%), Desulfobacterota (6.6%), and Omnitrophota (6.2%) (Fig. 2A, Supplementary Fig. 2A), previously identified as dominant members of deep microbiomes, such as in Fennoscandian Shield [35, 36, 54, 55] and Russian [21] groundwaters. Most archaeal representatives (Fig. 2B, Supplementary Fig. 2B) were affiliated with the DPANN superphylum (10.6% of global representatives), Halobacteriota (1%), *Candidatus* Iainarchaeota (1%), and Thermoplasmatota (0.6%) phyla. These findings are consistent with the ubiquity and abundance of Patescibacteria and DPANN in subsurface environments [17, 28, 56] and corroborate their ecological significance in energy-limited settings.

The depth distribution of microbial phyla varied with Patescibacteria, Pseudomonadota (formerly known as Proteobacteria), and DPANN decreasing in prevalence compared to the Bacillota (formerly known as Firmicutes), Bacteroidota, and Chloroflexota that were dominant in the deepest aquifers (Fig. 2C). Patescibacteria were the dominant representatives in depth categories (i)–(ii) with relative abundances of 37% and 31%, respectively, while declining to 4% in category (iii). Co-occurrence network analysis from groundwaters of the Hainich Critical Zone Exploratory in Germany highlights the central role of Patescibacteria [57], often suggested to be associated in a reciprocal partnership with autotrophic taxa involved in nitrogen, sulfur, and iron cycling [57]. Combined with the results herein, these findings supported that the decreased abundance of Patescibacteria with depth may be explained by the decreasing carbon and energy availability in the deeper groundwaters coupled with the challenge of finding partners in the more sparsely occupied deep groundwaters.

The phylum with the second highest relative abundance of genome clusters was Pseudomonadota that had more equal relative abundances with depth at 14.7, 20.3, and 13.6% in depth category (i)–(iii), respectively. The DPANN superphylum was detected in depth category (i) with 8.6% such as at 70 mbsl in Äspö HRL and 85 mbsl in Alberta, and depth category (ii) with 3.6% at 1340 mbsl in Beatrix mine but not at depths >2000 mbsl. The superphylum DPANN corresponds to ultra-small symbiont cells [33], where some are fix carbon dioxide through a modified version of the reductive acetyl-CoA (Wood–Ljungdahl, WL) pathway [17]. Finally, the deeper samples featured members of phyla Chloroflexota, detected in hot springs, wastewater treatment systems, and deep-sea sediments [58]; Bacillota described as a dominant member across the deep terrestrial subsurface biome [19]; and Bacteroidota, another common group in groundwater aquifers [59]. This analysis showed that the deep biosphere is a complex and stratified ecosystem where microbial community composition varies with depth. Key groups such as Pseudomonadota were consistently present across depths, forming a core part of the subsurface community. In contrast, Patescibacteria and DPANN were more prevalent in shallower regions, likely due to the challenge of finding symbiotic partners [57] and/or critical interactions for their survival [60, 61]. Finally, deeper zones were dominated by, for example, Chloroflexota, Bacillota, and Bacteroidota, highlighting stratified adaptations to energy-limited conditions.

### Genomic properties of representative MAGs/SAGs with depth

Modeling the representative MAGs/SAGs genomic GC content gave a (weak) statistically significant positive relationship between deeper depths and increased GC content (*P*-value = 5.79 e^−12^, *R*^2^ = 0.0011; Supplementary Fig. 3). The distribution of GC content for the global dominant phyla across each depth category indicated lineages with slightly higher GC content in deeper ecosystems (Fig. 2D), which was in agreement with a previous study showing the same trend in the Fennoscandian Shield [36]. In addition, the dominant individual phyla that showed a significant positive correlation between GC content and depth (Supplementary Fig. 4) were Desulfobacterota (*P*-value = 1.08e^−5^, *R*^2^ = 0.007), Bacillota_B (*P*-value = 2.2e^−16^, *R*^2^ = 0.2509), Methanobacteriota (*P*-value = 4.58e^−5^, *R*^2^ = 0.5063), and Patescibacteria (*P*-value = 3.13e^−9^, *R*^2^ = 0.0023). Among these, Bacillota_B and Methanobacteriota exhibited the strongest correlations. In contrast, Bacillota_E, Bacillota_G, Bacteroidota, Chloroflexota, Omnitrophota, Pseudomonadota, and DPANN showed no clear relationship between GC content and depth. Notably, Nitrospirota had a significant negative relationship, showing a GC content decrease with increasing depth (*P*-value = 1.13e^−8^, *R*^2^ = 0.0862). This suggested that deep groundwater populations may employ contrasting evolutionary strategies in response to the availability of nutrient and energy resources.

### Novel global representative MAGs/SAGs

Novel global representatives were identified based on the GTDB classification. These included 8 bacterial and 0 archaeal classes, 40 and 1 orders, 98 and 6 families, 361 and 85 genera, and 829 and 186 species, respectively (Supplementary Fig. 1B). The novel candidate taxa included a wide range of phyla, covering the relatively abundant Patescibacteria, Pseudomonadota, and DPANN (Supplementary Fig. 2 and Supplementary Table 5). This high proportion of previously unknown populations highlights the deep biosphere as a rich source of novel microorganisms [35]. However, further phylogenomic analysis will be necessary to confirm these candidates as valid novel taxa.

### Diversity indices reveal environmental influences on the core community

**Figure 3:**
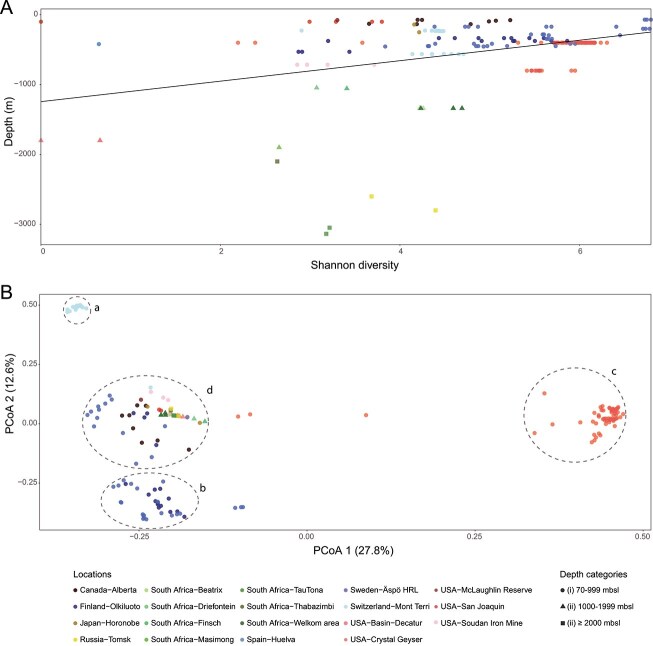
Diversity of global deep biosphere metagenomes. Shannon diversity index versus sampling depth (A) and PCoA of the Bray-Curtis index for global groundwater metagenomic datasets (B). The PCoA displays four MAGs/SAGs clusters (*a* to *d*); depth is categorized in three ranges: 70–999 mbsl (●), 1000–1999 mbsl (▲), and ≥2000 mbsl (■); and the sample location shown by the symbol color.

Shannon’s diversity of the global deep biosphere groundwaters plotted against depth (Fig. 3A, Supplementary Fig. 5, and Supplementary Table 6) showed Äspö HRL groundwaters in depth category (i) had the highest alpha diversity and species richness (6.41–6.79 and 629–921, respectively) followed by Crystal Geyser samples (6.23–6.30 and 529–574). A linear regression analysis indicated a statistically significant negative relationship between depth and Shannon diversity (*P-*value = 4.45e^−07^), agreeing with previous reports of decreasing diversity with depth [36, 54, 62, 63] linked to the decreasing organic carbon content [64] (it should also be noted that the sampling sites with the highest diversity were those with the greatest sequencing depth), or reduced cross-feeding options [36]. However, the model only explained ~14% of the variability in Shannon diversity (*R*^2^ = 0.14) and communities with low diversity and richness were identified in groundwaters in all three depth categories, suggesting that while depth is an important factor, other environmental variables likely contribute to the variance.

**Figure 4:**
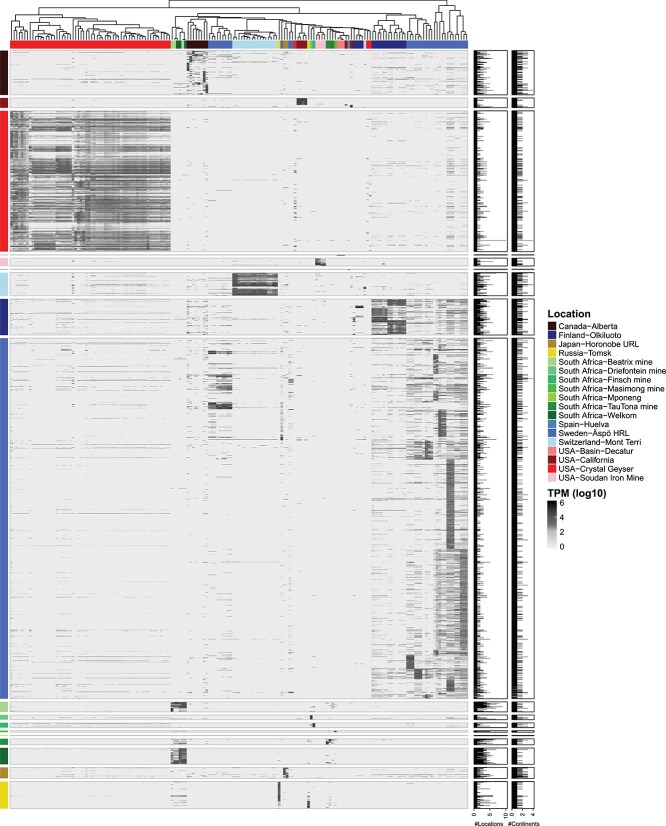
Heatmap showing metagenome read mapping against global deep representative genomes. The average abundance (log_10_ of TMP) of global deep groundwater representatives from metagenomes. Both metagenomes and global representatives are color-coded according to sampling origin. On the right, the number of metagenomes, locations, and continents where each representative has been identified is indicated based on average abundance.

A Principal Coordinates Analysis (PCoA) of the beta diversity showed community composition variation across locations and depths (Fig. 3B), with the first two axes explaining 27.8% and 12.6% of the variance, respectively. Four distinct MAGs/SAGs clusters (Fig. 3B) were observed for samples from Mont Terri (a), the Fennoscandian Shield (b), Crystal Geyser (c), and groundwater samples from 18 of the 19 global locations (d). The observed variation in microbial community composition was likely driven by environmental factors such as the clay-based Opalinus Clay rock borehole that was injected with hydrogen as electron donor [5] differing from the unadulterated granitic Fennoscandian Shield bedrock of varying ages and geochemistry’s [17]. In addition, MAGs/SAGs cluster (c) reflected microbial communities from multiple stratified sandstone aquifers [30, 33], likely influenced by the saturation of CO_2_ from the Crystal Geyser eruptions. The representative MAGs/SAGs distribution in different metagenomes also showed that Crystal Geyser representatives formed a distinct cluster with populations that were scarce in other global samples (Fig. 4). Finally, despite the diverse environmental conditions represented in the samples, MAGs/SAGs cluster (d) suggested a degree of overlap in microbial communities across different continents and depths, further supporting the hypothesis of a core microbial population present throughout the deep biosphere.

### Major contamination in deep biosphere datasets

A total of 51 out of 2255 microbial deep groundwater representatives (2.4%) were identified as potential common deep biosphere contaminants, as previously reported [50]. These contaminants were predominantly from the genera *Brevundimonas* (9 representatives), *Pseudomonas* (8), *Bradyrhizobium* (3), *Sphingobium* (3), alongside 19 other genera (Supplementary Table 7). In addition, the microbial population *Cutibacterium acnes* was detected in 10 geographically diverse locations, including Alberta-Canada, Crystal Geyser, Soudan iron mine, and San Joaquin Valley-USA, Olkiluoto Island-Finland, Mont Terri-Switzerland, Huelva-Spain, Tomsk-Russia, Thabazimbi and TauTona-South Africa, spanning all three depth categories (85–3048 mbsl; Supplementary Table 7). *C. acnes* is a Gram-positive, lipophilic microorganism and a dominant member of skin microbiota, commonly found in sebaceous, lipid-rich areas of human skin [65]. Despite being a human-related microorganism, its repeated detection across multiple deep groundwater sites raises the possibility of either genuine environmental persistence or contamination. However, given the typically ultra-low biomass of deep groundwater samples [66], the risk of contamination during drilling, sampling, or DNA extraction is significantly increased. In addition, *C. acnes* has a close relationship to the genus *Propionibacterium*, previously identified as a common skin-associated contaminant [50]. Therefore, *C. acne* was classified as a potential contaminant in this study and removed from the analyses.

### Global core deep groundwater populations

**Figure 5:**
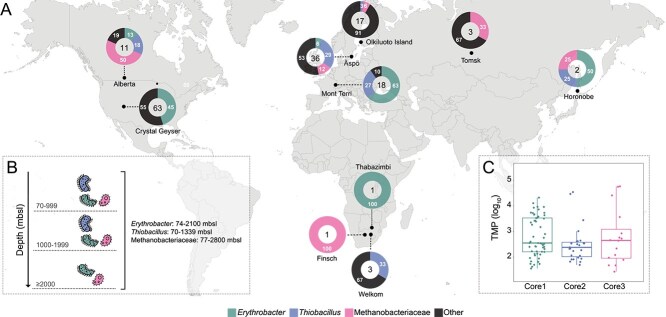
Global map displaying the core deep groundwater representatives identified in this study and their worldwide presence. The center of each donut plot (A) shows the total number of metagenomic samples from that location, while the donut segments represent the percentage of samples containing each global core deep groundwater representative. *Erythrobacter* is represented in dark cyan, *Thiobacillus* in blue, Methanobacteriaceae in pink, and other microbial populations in gray. In addition, shown are the presence of each global core representative at different sampling depths (B) and the average abundance of each global core representative (C).

None of the 2204 representative MAGs/SAGs in the metagenomes (threshold of log_10_ TPM >1 for normalization) were omnipresent across all 19 locations (Fig. 4). This was likely explained by the geochemical differences between the locations, such as varying depths and geological formation of each sampled groundwater along with differences in sequencing depth capturing varying degrees of the microbial community diversity. Additionally, the dataset is skewed toward shallower environments, with limited representation from depth category (iii). This may bias the recovery of core taxa toward organisms adapted to these conditions. As more (meta)genomic datasets become available from deeper environments, the detection of truly ubiquitous populations may increase. Despite this, it was possible to identify a set of common core deep groundwater representatives seen across all four continents (America, Europe, Asia, and Africa). These common core deep groundwater representatives (Fig. 5A) included genome clusters affiliated to bacterial genera *Erythrobacter* sp002842735 (MAG CA1_SRR13727520.4) and *Thiobacillus* sp002256995 (MAG SweH_Old_saline_planktonic.mb.76), and the archaeal family Methanobacteriaceae UBA349 sp023249725 (MAG CA7_SRR13727535.4), that will be referred to as *Erythrobacter*, *Thiobacillus*, and Methanobacteriaceae, respectively. These three populations were identified in 10 of the 19 locations, with Alberta, Horonobe, and Äspö HRL being the only locations hosting all three. Out of the 174 metagenomes analyzed, 81 included at least one global core population (46.6% of the total metagenomic dataset). These three populations were found at depths ranging from 70 to 2800 mbsl (Fig. 5B) with abundances ranging from 1.55 to 4.32 log_10_ TPM for *Erythrobacter*, 1.69–4.54 for *Thiobacillus*, and 1.43–4.76 for Methanobacteriaceae (Fig. 5C).

The *Erythrobacter* population was identified in six countries (Alberta-Canada, Crystal Geyser-USA, Mont Terri-Switzerland, Äspö HRL-Sweden, Honorobe URL-Japan, and Thabazimbi-South Africa) and across all three depth categories (74–2100 mbsl; Supplementary Table 8). Members of the *Erythrobacter* genus (Alphaproteobacteria) typically inhabit marine surface environments and are capable of producing pigments, with some species also capable of producing bacteriochlorophyll [67]. This genus is reported as highly abundant in microbial ecosystems from deep-sea sediments ranging from 1681 to 2409 m in the southern Colombian Sea [68] and in samples collected at depths of 5–2700 m in the South Atlantic Ocean [69].

The *Thiobacillus* genus core population was identified in six locations (Alberta-Canada, Olkiluoto Island-Finland, Äspö HRL-Sweden, Mont Terri-Switzerland, Honorobe URL-Japan, and Welkom-South Africa) between 70 and 1339 mbsl in depth categories (i) and (ii) (Supplementary Table 8). This Betaproteobacterial lineage comprises sulfur-driven autotrophic denitrifiers that are dominant and active members in a fault zone at 1.34 km depth in Witwatersrand Basin, South Africa [8, 46]. They also play key roles in old groundwater obtained from confined aquifers in Canada (<250 m depth) [26], as a biofilm former in groundwater at 448 mbsl from Äspö HRL [55], and *Thiobacillus denitrificans* is present in MAGs and as SSU rRNA transcripts from old saline Äspö HRL groundwater [9, 70].

The Methanobacteriaceae population was detected in samples from Alberta-Canada, Olkiluoto Island-Finland, Äspö HRL-Sweden, Honorobe URL-Japan, Tomsk-Russia, and Finsch-South Africa at depths from 77 to 2800 mbsl (i.e. all three depth categories; Supplementary Table 8). The family Methanobacteriaceae (UBA349) are methanogenic archaea, which have been detected in deeper artesian water collected at a 2.8 km borehole 5P in Russia [21], in reconstructed MAGs/SAGs from the Fennoscandian Shield [17], and in a MAG reconstructed from old groundwaters in Canada [26].

The Bacillota *Candidatus* Desulforudis audaxviator was originally discovered at 2.8 km depth in a South African gold mine. It is described as a motile, sporulating, chemoautotrophic thermophile capable of nitrogen and carbon fixation [24]. This sulfate-reducing bacteria is found in geographically widespread deep groundwater locations across three continents: Africa (Mponeng, Beatrix and TauTona), North America (borehole Inyo-BLM 1), and Eurasia (borehole BY-1R, West Siberia) [25]. Additionally, a recent publication revealed that *Candidatus* Desulforudis audaxviator dominated the COSC-2 groundwater borehole at a depth of 975 m in the Fennoscandian Shield [71]. In this study’s dataset, *Candidatus* Desulforudis audaxviator MP104C was identified in TauTona, Beatrix, Masimong, Thabazimbi, and the Welkom area in South Africa, as well as in Tomsk, Russia, confirming previous findings. Given this background and previous evidence confirming its presence across the four continents, *Candidatus* Desulforudis audaxviator MP104C was proposed as the fourth representative of the global deep groundwater microbiome.

### Metabolism potential in core deep groundwaters microbial populations

**Figure 6:**
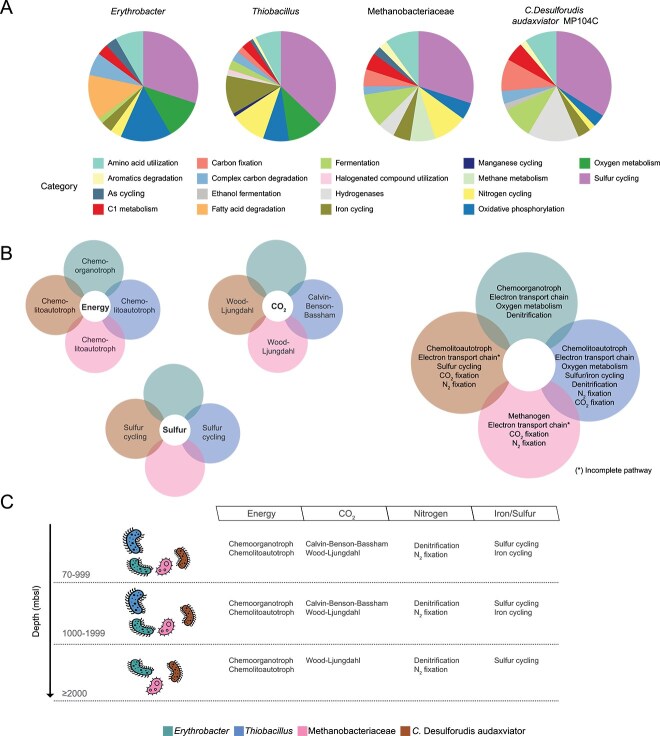
Metagenomic traits of the global core deep groundwater genomes. METABOLIC-C based metabolic traits in the four global core deep groundwater representative genomes (A), Venn diagrams showing the common and distinct metabolic traits among the five global core deep groundwater representatives (B), and metabolic traits at different sampling depths (C).

The energy and nutrient availability in the deep biosphere is typically limited and can originate from either geogenic or biogenic sources [72]. Global core deep groundwater representatives exhibit diverse metabolic properties, reflecting variations in energy flow, environmental adaptability, and metabolic complexity (Fig. 6A and B, Supplementary Table 9).

Even if the deep biosphere is frequently scarce in bioavailable organic carbon, heterotrophic microorganisms are identified [73] and likely utilize metabolic intermediates or biogenic products from chemolithoautotrophs. For instance, the presence of genes coding for amylolytic enzymes (beta-glucosidase *bglX* and alpha-amylase *treS*), chitin-degrading enzymes (beta-N-acetylhexosaminidase *nagZ*), and fatty acid-degrading enzymes (acyl-CoA dehydrogenase *acd* and acyl-CoA dehydrogenase *aidB*) in the *Erythrobacter* core group implies a chemoorganotroph lifestyle [74] via the breakdown of polysaccharides such as cellulose and chitin. Additionally, the presence of acyl-CoA dehydrogenase (*acd*) supported *Erythrobacter*’s capacity for fatty acid degradation. The *Erythrobacter* core group also coded for electron transport chain components with *nuoABC* genes for the NADH oxidoreductase (Complex I), *sdhCD* for subunits of succinate dehydrogenase (Complex II), and *petAB* coding for cytochrome *bc*_1_ complex components (Complex III). They also presented genes for aerobic respiration (e.g. a complex IV *caa*_3_-type cytochrome *c* oxidase), components of a *cbb*_3_-type cytochrome *c* oxidase (*ccoNOP*) mainly expressed under microaerobic conditions [75], *cydAB* encoding a cytochrome *bd* oxidase adapted to function at low oxygen tensions [76], and denitrification genes including nitrate reductase *narGH,* suggesting it can sustain respiration at varying oxygen levels. Finally, the *atpAD* genes encode subunits of the ATP synthase (Complex V). The core *Erythrobacter* was identified in the shallower depth categories (i) and (ii), where it may play a role in organic carbon infiltration either from the surface [77] or fixed carbon by autotrophic members of the community in the form of necromass [35].

*Thiobacillus* species are typically autotrophic [46, 78] and the core genome group contained key genes for the Calvin–Benson–Bassham (CBB) cycle, including *cbbLM* for 1,5-biphosphate carboxylase/oxygenase (RuBisCO). The *Thiobacillus* core group also encoded genes for chemolithotrophy, such as the sulfide oxidizing sulfide:quinone oxidoreductase (*sqr*), sulfur oxidizing Sox protein subunits (*soxBY*), sulfur dioxygenase (*sdo*), and adenylylsulfate reductase (*aprA*) suggesting it can oxidize sulfur and sulfur compounds [72]. Sulfur oxidation can be linked to electron transport with genes encoding components of NADH-quinone oxidoreductase (*nuoABC*, Complex I), subunits of succinate dehydrogenase (*sdhD*, Complex II), components of the cytochrome *bc*_1_ complex (*petAB*, Complex III), and subunits of the ATP synthase (*atpAD*, Complex V). In addition, the *Thiobacillus* genome encoded *caa*_3_-type cytochrome *c* oxidase (*coxAB*) subunits and *ccoNOP* expressed under aerobic [75] and microaerobic conditions [75], respectively; *cydAB* genes for a cytochrome *bd* oxidase adapted to function at low oxygen [76], and denitrification genes including nitrate reductase (*narGH*), nitrite reductase *nirBDS*, nitric oxide reductase (*norBC*); and *nifDKH* subunits of the nitrogenase enzyme complex. Furthermore, the *Thiobacillus* genome contained genes coding for ferric iron reduction (*dmkAB*, *fmnB*, *ndh2*, and *eetAB*) and dissimilatory sulfate reduction components *dsrAB* and *sat*. Sulfur cycling between sulfur oxidizers and sulfate reducers is an important metabolic process in the deep biosphere [38], and the presence of *Thiobacillus* in the core community suggests that it may play a central role in this cycle.

The methanogenic Methanobacteriaceae [21, 26] core group genome contained *mcrABC* genes that encode the methyl-coenzyme M reductase complex catalyzing the final step in methanogenesis alongside Group 3c (*mvhA*) and 4hi (*EHBn*) Ni-Fe hydrogenases that facilitate electron flow from reduced ferredoxins and mediate H₂ oxidation. This supported both methane production and anaerobic metabolism through reverse methanogenesis, suggesting that this group may engage in anaerobic methane oxidation. In addition, *atpAB* genes typically associated with complex V were identified, potentially suggesting a specialized or modified use of these genes involved in electron transfer processes beyond those associated with respiration. A similar dual role has been proposed for the V-type ATP synthase in the Thaumarchaeota genus *Candidatus* Nitrosotalea, where it can be involved in both ATP synthesis and proton export [79]. Interestingly, the Methanobacteriaceae UBA349 sp023249725 (MAG CA7_SRR13727535.4) core possesses *cdhDE* genes, which encode for acetyl-CoA decarbonylase/synthase subunits involved in the WL pathway. Although the primary mode of metabolism for *Methanobacteria* is methanogenesis, an incomplete WL pathway might serve a secondary function related to energy conservation or CO₂ assimilation with the coupling of methanogenesis and WL being considered one of the most ancient metabolisms for energy generation and carbon fixation in Archaea [80]. Finally, the nitrogenase (*nifH*) involved in nitrogen fixation was identified in the Methanobacteriaceae. Cell abundance decreases with depth, temperature, and ionic strength in the continental subsurface [13], while higher metabolic rates and biomass generation are observed for methanogens [15]. This implies that the core Methanobacteriaceae family plays a role in carbon fixation in all three depth categories (Fig. 6C).

Cultured representatives of *Candidatus* Desulforudis audaxviator are described as using organic carbon compounds and hydrogen coupled to sulfate reduction while fixing carbon via the WL pathway [81]. In accordance with the previous description, the core genome harbored genes for the WL pathway (Fig. 6B and C), including acetyl-CoA decarboxylase/synthase (*cdhDE*) and anaerobic carbon-monoxide dehydrogenase (*cooS*). Additionally, a molybdenum-iron protein encoded by *nifD* was identified, suggesting a nitrogen fixation mechanism. The core genome also contained genes coding for NiFe-group-1 and 4ag (*hyaB*, *echE*) as well as FeFe-group-a4, a13, and b (*hndD*), suggesting the oxidation and production of hydrogen, respectively. Energy conservation was suggested to be via the dissimilatory sulfate-reduction sulfite reductase subunits (*dsrABCDJ*), sulfate adenylyltransferase (*sat*), and the electron transport Complex V *atpAD* genes. *Candidatus* Desulforudis audaxviator has been identified in the deep biosphere on several continents [24, 71, 81]. This suggests its oxidation of organic carbon plus hydrogen that may be provided by geogenic origin, or a product of fermentation, may be an important growth strategy in these low carbon and energy groundwaters.

### Global conservation and genomic stability

**Figure 7:**
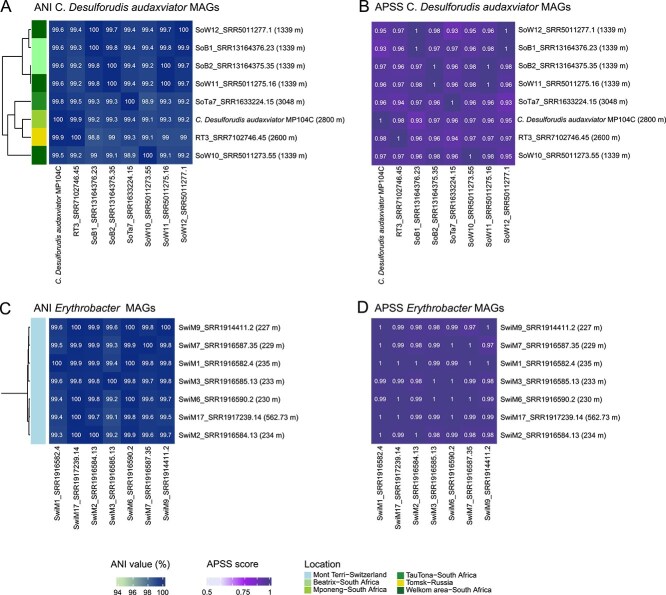
Genome comparison of global core deep groundwater genomes. Representative *Candidatus* Desulforudis audaxviator and *Erythrobacter* average nucleotide identities (A, C) and average pairwise synteny score (B, D) (*n* = 8 for both representatives) compared to other reconstructed MAGs and SAGs. Each reconstructed genome is color-coded according to its sampling origin.

A previous study compared two cultivated strains of *Candidatus* Desulforudis audaxviator: strain BYF from a 2 km-deep aquifer in Western Siberia and MP104C from South Africa. Genomic comparisons between these strains indicate remarkable genetic similarity, with an ANI of 99.95%, suggesting minimal evolutionary divergence despite geographic separation [81]. These findings imply that *Candidatus* Desulforudis audaxviator exhibits genomic stability and adaptability, enhancing our understanding of the evolutionary dynamics of subsurface microorganisms. In this analysis, seven MAGs associated with the *Candidatus* Desulforudis audaxviator taxonomic group were identified from the Beatrix, Mponeng, TauTona, Welkom areas in South Africa, as well as from Alberta (Canada), Mont Terri (Switzerland), and Tomsk (Russia). The SAG *Candidatus* Desulforudis audaxviator MP104C from Mponeng was included and selected as a representative genome for this group. The global *Candidatus* Desulforudis audaxviator representatives exhibited an average ANI of 99.48 ± 0.34% (Fig. 7A) and an Average Pairwise Synteny Scores (APSS) of 0.96 ± 0.02 (Fig. 7B). The minimum ANI was 98.83% between the Tomsk and Beatrix genomes, while the maximum was 100% among the South Africa genomes. The minimum and maximum synteny scores were 0.93 between the South African Welkom area and TauTona genomes compared with 1 for the Welkom area and Beatrix genomes. The high identity and synteny scores of *Candidatus* Desulforudis audaxviator genomes from various groundwater sources in Africa and Asia supported the previous study of minimal evolution since the physical separation of deep borehole fluids in Africa (Kaapvaal craton), sedimentary rocks in North America and Eurasia [15, 71], and Swedish imbricates of sandstone and conglomerates [71].

A similar analysis was conducted for *Erythrobacter*, utilizing seven genomes from Mont Terri, yielding an average ANI of 99.7 ± 0.24 (Fig. 7C) and an average synteny score of 0.99 ± 0.01 (Fig. 7D). The ANI values ranged from a minimum of 99.1% to a maximum of 100%, supported by high synteny scores between 0.97 and 1. The elevated ANI and APSS values for *Erythrobacter* may be attributed to the environmental similarity within Mont Terri. Additionally, 11 populations with higher numbers of reconstructed MAGs (not global core populations), located across two or three continents, were analyzed using ANI (Supplementary Fig. 6), while six populations were examined for APSS synteny comparison values (Supplementary Fig. 7). These results suggest that global populations presented higher ANI percentages (>98%–100%) such as the core *Candidatus* Desulforudis audaxviator and *Erythrobacter*, which may be exclusive to the deep biosphere.

## Conclusions

This study identified four global deep groundwater populations; namely *Erythrobacter*, *Thiobacillus*, Methanobacteriaceae, and *Candidatus* Desulforudis audaxviator, at varying depths across four continents. Based on the global deep biosphere datasets from this research, the core deep microbial community in the first two depth categories (70–1999 mbsl) consisted of chemoorganotrophic microorganisms alongside chemolithoautotrophic representatives utilizing CBB and WL pathways for carbon fixation, with the rTCA cycle notably absent. At greater depths, the core deep community transitioned to methanogens and chemolithoautotrophs, primarily relying on the WL pathway for carbon fixation. Finally, comparison of genomes from the same species at different locations suggested minimal evolutionary divergence for *Erythrobacter* and *Candidatus* Desulforudis audaxviator across multiple continents since their separation from a common ancestral population.

## Supplementary Material

Supplementary_figure_1_ycaf176

Supplementary_figure_2_ycaf176

Supplementary_figure_3_ycaf176

Supplementary_figure_4_ycaf176

Supplementary_figure_5_ycaf176

Supplementary_figure_6_ycaf176

Supplementary_figure_7_ycaf176

Supplementary_Table_1_ycaf176

Supplementary_Table_2_ycaf176

Supplementary_Table_3_ycaf176

Supplementary_Table_4_ycaf176

Supplementary_Table_5_ycaf176

Supplementary_Table_6_ycaf176

Supplementary_Table_7_ycaf176

Supplementary_Table_8_ycaf176

Supplementary_Table_9_ycaf176

## Data Availability

The MAG and SAGs generated in this study are publicly available in figshare under the project “Deep biosphere populations” with the identifier http://dx.doi.org/10.6084/m9.figshare.28190006.v1. The 2255 global deep biosphere representatives are publicly available in figshare under the project “Deep biosphere representatives” with the identifier http://dx.doi.org/10.6084/m9.figshare.28190012.v1. All data supporting the findings of this article are available within this article and its supplementary material. All the programs used, as well as the version and set threshold, are mentioned in the manuscript and supplementary information. A compiled version of the R Markdown document with the bioinformatic pipeline are provided on figshare under the project “Deep biosphere R Markdown” with the identifier http://dx.doi.org/10.6084/m9.figshare.28190024.v1.
