# Supplementary figures and images for: A global deep terrestrial biosphere core microbiome
Source: ISME Commun. 2025 Oct 7;5(1):ycaf176. doi: 10.1093/ismeco/ycaf176 (PMC12596165; doi:10.1093/ismeco/ycaf176)

a

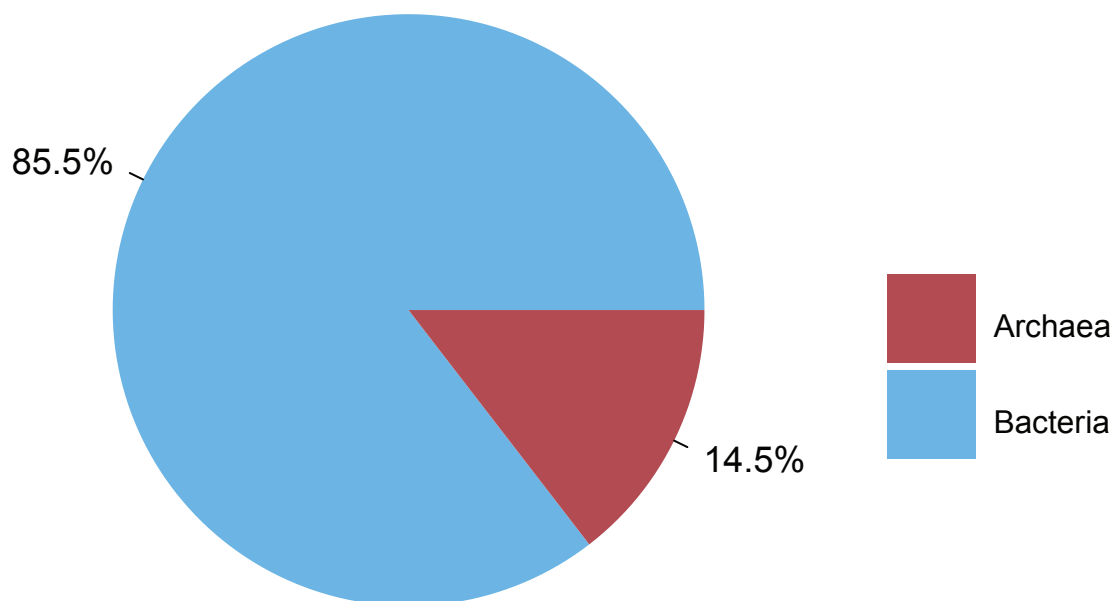

b

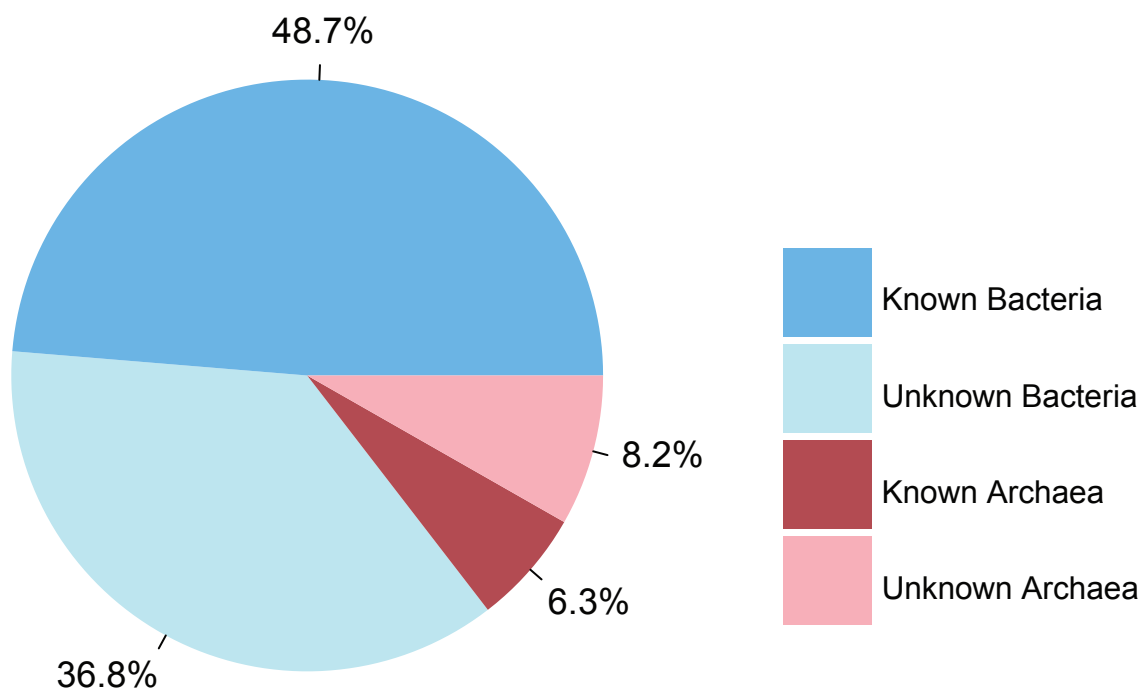

Supplement: Supplementary_figure_1_ycaf176 [file supplementary_figure_1_ycaf176.pdf]

a

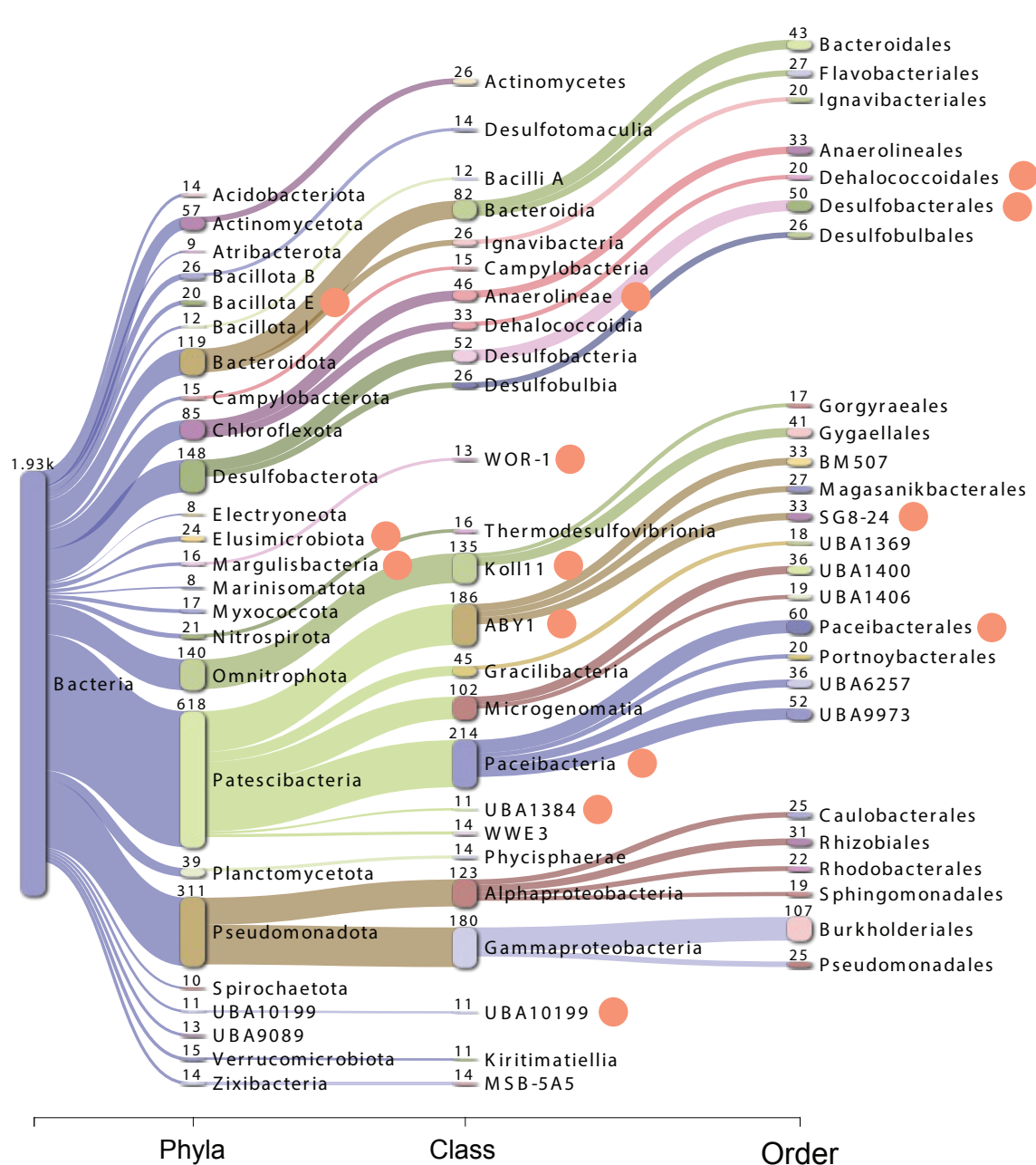

b

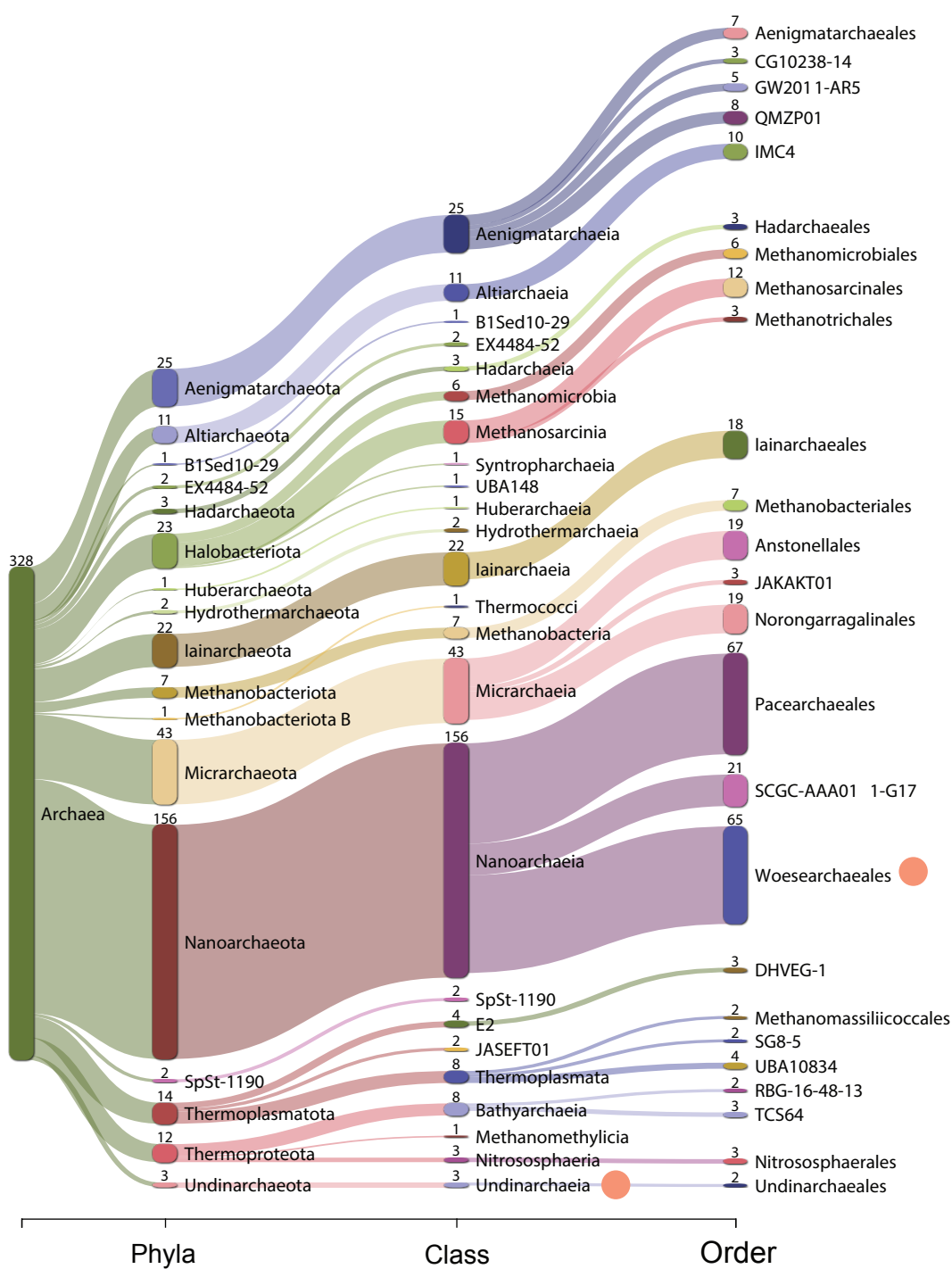

Supplement: Supplementary_figure_2_ycaf176 [file supplementary_figure_2_ycaf176.pdf]

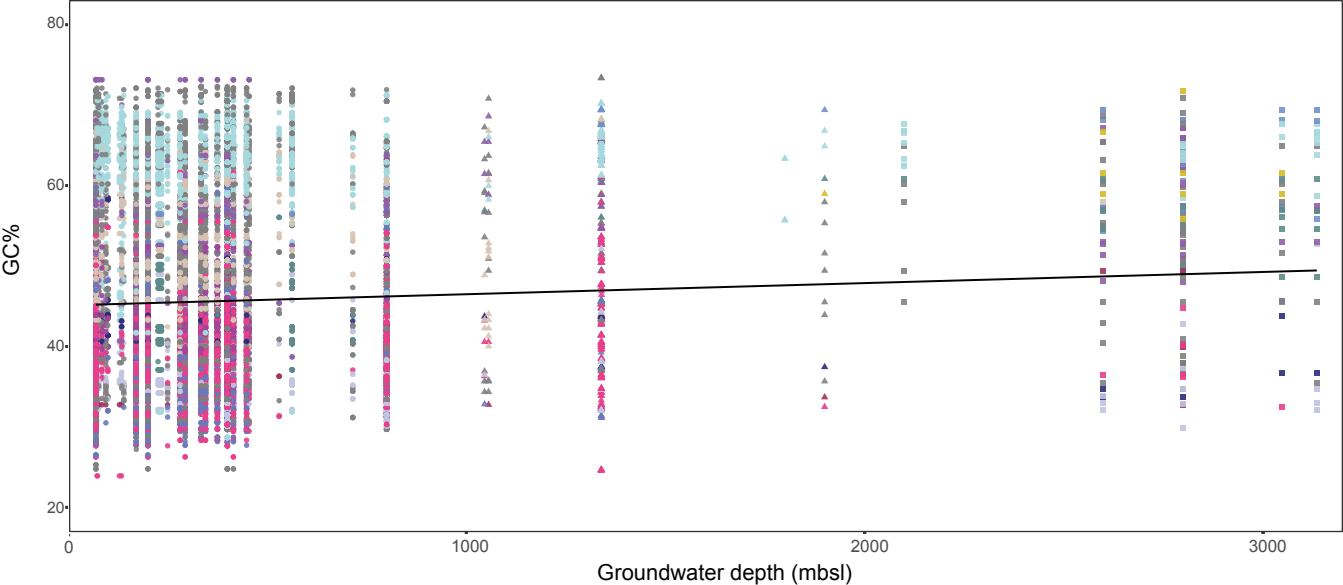

Supplement: Supplementary_figure_3_ycaf176 [file supplementary_figure_3_ycaf176.pdf]

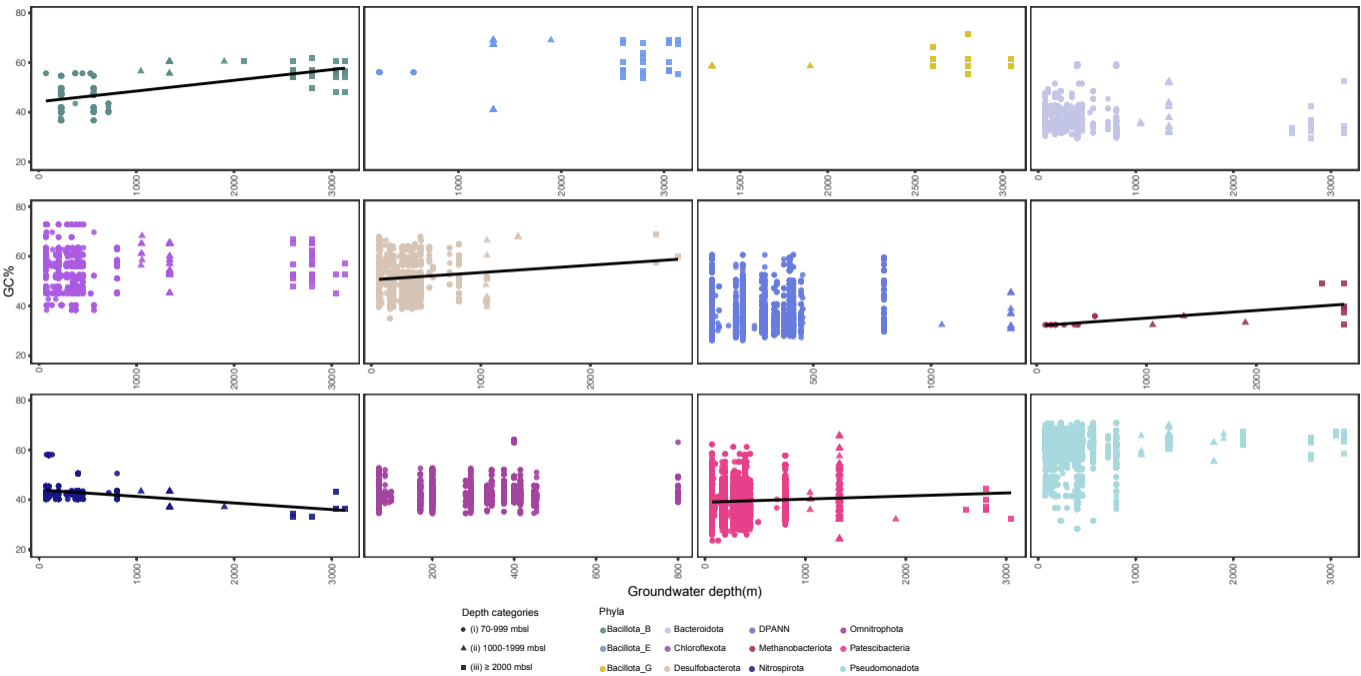

Supplement: Supplementary_figure_4_ycaf176 [file supplementary_figure_4_ycaf176.pdf]

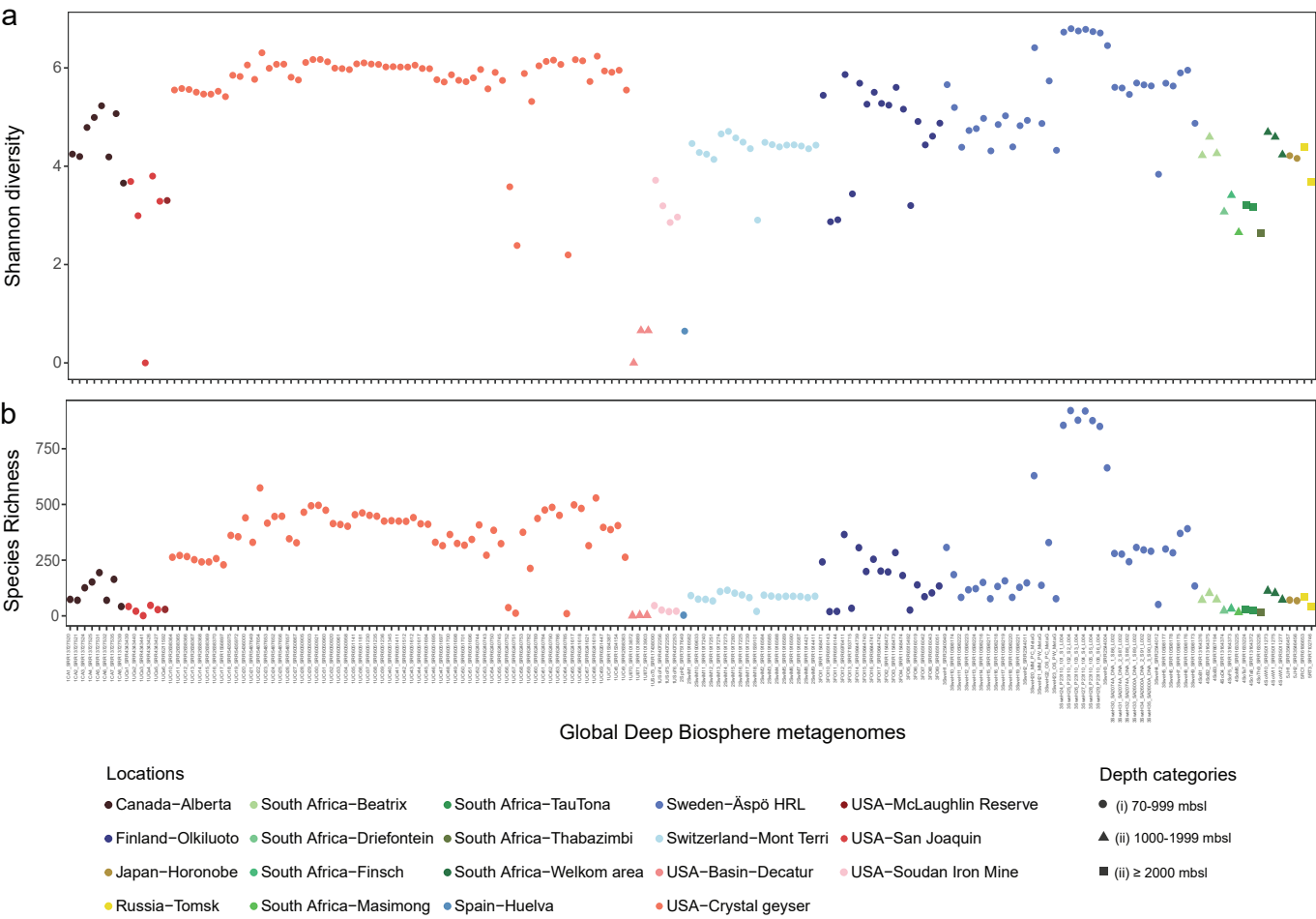

Supplement: Supplementary_figure_5_ycaf176 [file supplementary_figure_5_ycaf176.pdf]
