# Supplementary material for: A global deep terrestrial biosphere core microbiome
Source: ISME Commun. 2025 Oct 7;5(1):ycaf176. doi: 10.1093/ismeco/ycaf176 (PMC12596165; doi:10.1093/ismeco/ycaf176)
Supplement: Supplementary_figure_6_ycaf176 [file supplementary_figure_6_ycaf176.pdf]

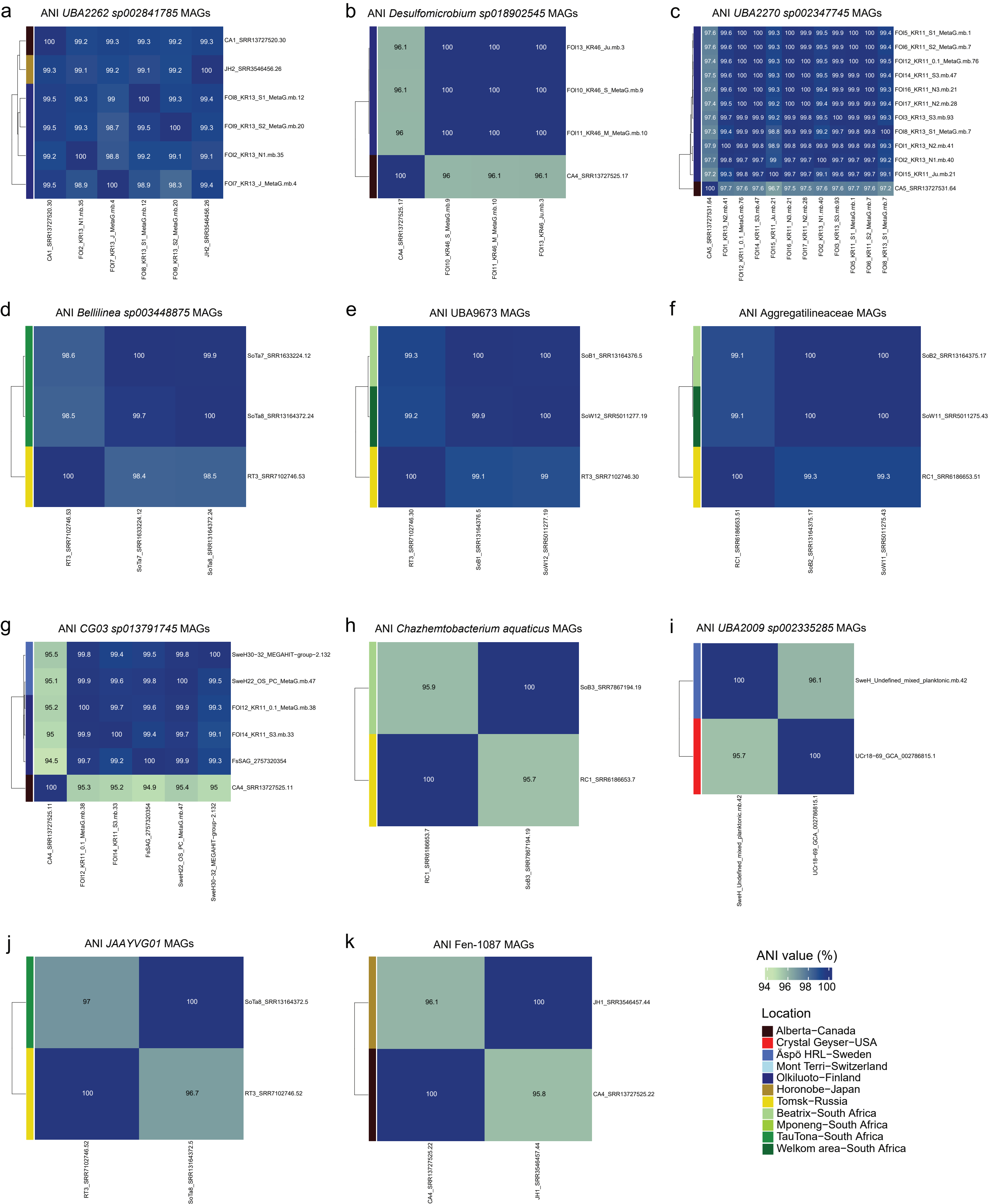
a
ANI UBA2262 sp002841785 MAGs
CA1_SRR13727520.30
JH2_SRR3546456.26
FOI8_KR13_S1_MetaG.mb.12
FOI9_KR13_S2_MetaG.mb.20
FOI2_KR13_N1.mb.35
FOI7_KR13_J_MetaG.mb.4
b
ANI Desulfomicrobium sp018902545 MAGs
FOI13_KR46_Ju.mb.3
FOI10_KR46_S_MetaG.mb.9
FOI11_KR46_M_MetaG.mb.10
CA4_SRR13727525.17
c
ANI UBA2270 sp002347745 MAGs
FOI5_KR11_S1_MetaG.mb.1
FOI6_KR11_S2_MetaG.mb.7
FOI12_KR11_0.1_MetaG.mb.76
FOI14_KR11_S3.mb.47
FOI16_KR11_N3.mb.21
FOI17_KR11_N2.mb.28
FOI3_KR13_S3.mb.93
FOI8_KR13_S1_MetaG.mb.7
FOI1_KR13_N2.mb.41
FOI2_KR13_N1.mb.40
FOI15_KR11_Ju.mb.21
CA5_SRR13727531.64
d
ANI Bellilinea sp003448875 MAGs
SoTa7_SRR1633224.12
SoTa8_SRR13164372.24
RT3_SRR7102746.53
e
ANI UBA9673 MAGs
SoB1_SRR13164376.5
SoW12_SRR5011277.19
RT3_SRR7102746.30
f
ANI Aggregatilineaceae MAGs
SoB2_SRR13164375.17
SoW11_SRR5011275.43
RC1_SRR6186653.51
g
ANI CG03 sp013791745 MAGs
SweH30−32_MEGAHIT−group−2.132
SweH22_OS_PC_MetaG.mb.47
FOI12_KR11_0.1_MetaG.mb.38
FOI14_KR11_S3.mb.33
FsSAG_2757320354
CA4_SRR13727525.11
h
ANI Chazhemtobacterium aquaticus MAGs
SoB3_SRR7867194.19
RC1_SRR6186653.7
i
ANI UBA2009 sp002335285 MAGs
SweH_Undefined_mixed_planktonic.mb.42
UCr18−69_GCA_002786815.1
j
ANI JAAYVG01 MAGs
SoTa8_SRR13164372.5
RT3_SRR7102746.52
k
ANI Fen-1087 MAGs
JH1_SRR3546457.44
CA4_SRR13727525.22
ANI value (%)
94 96 98 100
Location
Alberta−Canada
Crystal Geyser−USA
Äspö HRL−Sweden
Mont Terri−Switzerland
Olkiluoto−Finland
Horonobe−Japan
Tomsk−Russia
Beatrix−South Africa
Mponeng−South Africa
TauTona−South Africa
Welkom area−South Africa
