# Supplementary material for: A global deep terrestrial biosphere core microbiome
Source: ISME Commun. 2025 Oct 7;5(1):ycaf176. doi: 10.1093/ismeco/ycaf176 (PMC12596165; doi:10.1093/ismeco/ycaf176)
Supplement: Supplementary_figure_7_ycaf176 [file supplementary_figure_7_ycaf176.pdf]

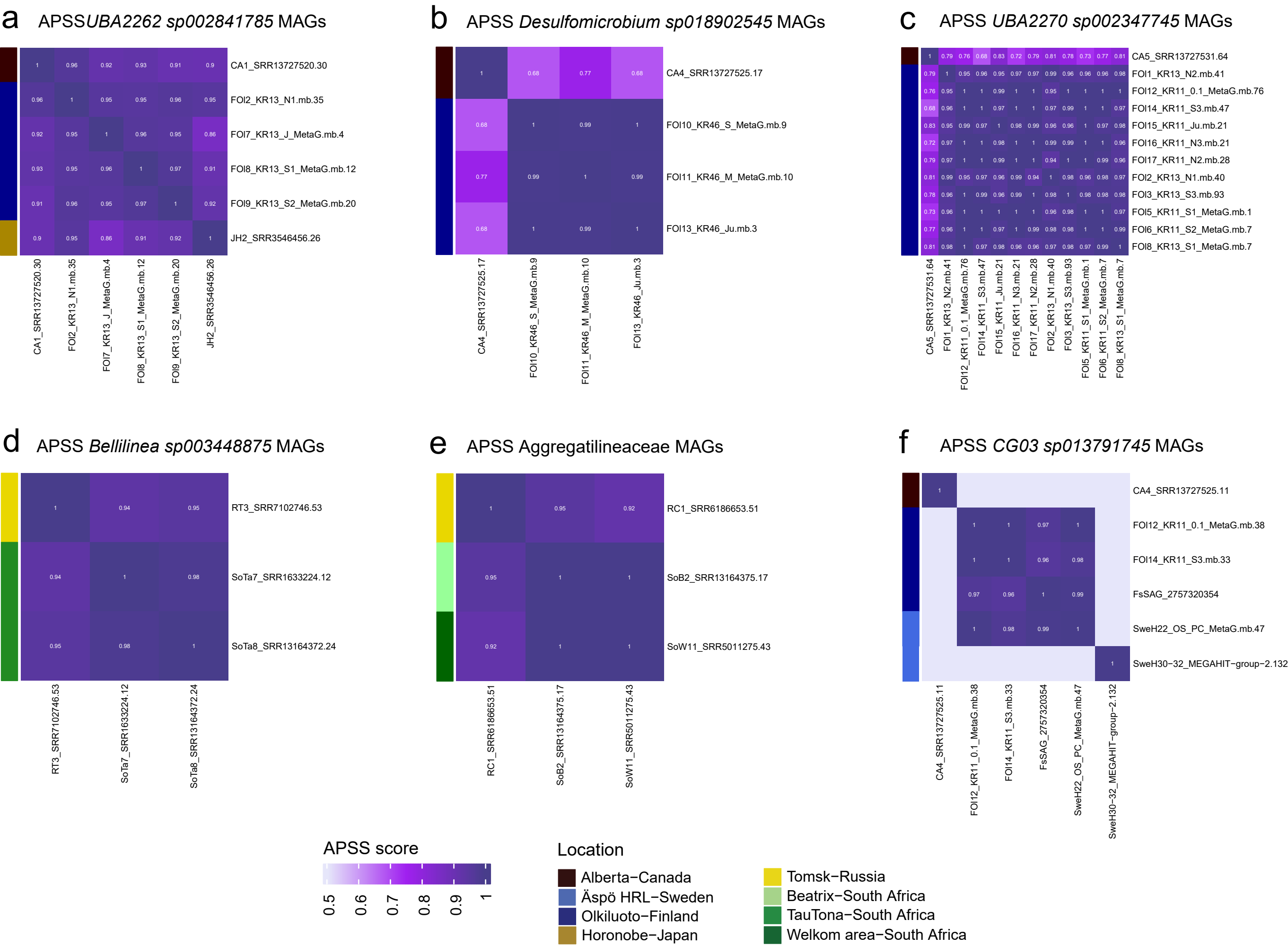
a
APSS UBA2262 sp002841785 MAGs
CA1_SRR13727520.30
FOI2_KR13_N1.mb.35
FOI7_KR13_J_MetaG.mb.4
FOI8_KR13_S1_MetaG.mb.12
FOI9_KR13_S2_MetaG.mb.20
JH2_SRR3546456.26
b
APSS Desulfomicrobium sp018902545 MAGs
CA4_SRR13727525.17
FOI10_KR46_S_MetaG.mb.9
FOI11_KR46_M_MetaG.mb.10
FOI13_KR46_Ju.mb.3
c
APSS UBA2270 sp002347745 MAGs
CA5_SRR13727531.64
FOI1_KR13_N2.mb.41
FOI12_KR11_0.1_MetaG.mb.76
FOI14_KR11_S3.mb.47
FOI15_KR11_Ju.mb.21
FOI16_KR11_N3.mb.21
FOI17_KR11_N2.mb.28
FOI2_KR13_N1.mb.40
FOI3_KR13_S3.mb.93
FOI5_KR11_S1_MetaG.mb.1
FOI6_KR11_S2_MetaG.mb.7
FOI8_KR13_S1_MetaG.mb.7
d
APSS Bellilinea sp003448875 MAGs
RT3_SRR7102746.53
SoTa7_SRR1633224.12
SoTa8_SRR13164372.24
e
APSS Aggregatilineaceae MAGs
RC1_SRR6186653.51
SoB2_SRR13164375.17
SoW11_SRR5011275.43
f
APSS CG03 sp013791745 MAGs
CA4_SRR13727525.11
FOI12_KR11_0.1_MetaG.mb.38
FOI14_KR11_S3.mb.33
FsSAG_2757320354
SweH22_OS_PC_MetaG.mb.47
SweH30-32_MEGAHIT-group-2.132
APSS score
0.5
0.6
0.7
0.8
0.9
1
Location
Alberta–Canada
Äspö HRL–Sweden
Olkiluoto–Finland
Horonobe–Japan
Tomsk–Russia
Beatrix–South Africa
TauTona–South Africa
Welkom area–South Africa
